# Discovery of a novel natural product inhibitor of *Clostridioides difficile* with potent activity *in vitro* and *in vivo*

**DOI:** 10.1371/journal.pone.0267859

**Published:** 2022-08-08

**Authors:** Rusha Pal, Mohamed N. Seleem

**Affiliations:** 1 Department of Comparative Pathobiology, College of Veterinary Medicine, Purdue University, West Lafayette, Indiana, United States of America; 2 Department of Biomedical Sciences and Pathobiology, Virginia-Maryland College of Veterinary Medicine, Virginia Polytechnic Institute and State University, Blacksburg, Virginia, United States of America; 3 Center for Emerging, Zoonotic and Arthropod-borne Pathogens, Virginia Polytechnic Institute and State University, Blacksburg, Virginia, United States of America; University of Illinois at Chicago, UNITED STATES

## Abstract

*Clostridioides difficile* infection is a global health threat and remains the primary cause of hospital-acquired infections worldwide. The burgeoning incidence and severity of infections coupled with high rates of recurrence have created an urgent need for novel therapeutics. Here, we report a novel natural product scaffold as a potential anticlostridial lead with antivirulence properties and potent activity both *in vitro* and *in vivo*. A whole cell phenotypic screening of 1,000 purified natural products identified 6 compounds with potent activity against *C*. *difficile* (minimum inhibitory concentration (MIC) range from 0.03 to 2 μg/ml). All these 6 compounds were non-toxic to human colorectal cells. The natural product compounds also inhibited the production of key toxins, TcdA and TcdB, the key virulence determinants of *C*. *difficile* infection pathology. Additionally, the compounds exhibited rapid bactericidal activity and were superior to the standard-of-care antibiotic vancomycin, in reducing a high inoculum of *C*. *difficile in vitro*. Furthermore, a murine model of *C*. *difficile* infection revealed that compound NP-003875 conferred 100% protection to the infected mice from clinical manifestations of the disease. Collectively, the current study lays the foundation for further investigation of the natural product NP-003875 as a potential therapeutic choice for *C*. *difficile* infection.

## 1. Introduction

*Clostridioides* (*Clostridium*) *difficile* is an opportunistic, Gram-positive, obligatory anaerobic, pathogenic bacillus that causes debilitating diarrhea and potentially life-threatening intestinal inflammation [1, 2]. In 2017 alone, this pathogen was responsible for approximately 223,900 hospitalizations that resulted in over 12,800 deaths in the United States, thus imposing a substantial health burden [3].

A healthy intestinal microbiome can provide colonization resistance to *C*. *difficile*. However, broad-spectrum antibiotics cause structural and functional alteration of the gut microbiota, thereby, generating an environment conducive to *C*. *difficile* spore germination and vegetative cell outgrowth [4, 5]. Inside the large intestine, the metabolically active cells secrete the two key virulence determinants of *C*. *difficile* infection (CDI), toxin A (TcdA) and toxin B (TcdB), the clinical sequelae of which ranges from mild or moderate diarrhea to severe complications characterized by fulminant colitis, toxic megacolon, sepsis, and even death [6, 7]. These pathogenic exotoxins cause GI inflammation to persist which favors an optimal niche for continued survival of the pathogen [8, 9]. Indeed, epidemiological and laboratory findings corroborate that only toxinogenic strains of *C*. *difficile*, typically producing both TcdA and TcdB, cause disease [10–12].

The therapeutic armamentarium for treating *C*. *difficile* infection (CDI) includes the antibiotics vancomycin and fidaxomicin. Metronidazole can be used for treating non-severe cases only in settings with limited access to vancomycin and fidaxomicin [13]. However, antibiotic intervention is not always successful as about 40–60% of the patients with prior infection experience recurrence within 3 weeks following the discontinuation of antibiotics [14]. Furthermore, neither vancomycin nor metronidazole has an inhibitory effect on toxin production by *C*. *difficile* [15]. With the impending loss of effective therapeutic options that can promote a sustained clinical resolution, new antimicrobial agents that can counteract the pathogen and its virulence factors are urgently needed.

Despite the dwindling interest of the pharmaceutical industry in natural product research over the past two decades, natural products continue to be a major source of lead compound identification for drug discovery [16]. Of the 1,881 new drugs that received approval by the U.S Food and Drug Administration (FDA) between 1981 and 2019, 546 drugs are either natural products or natural product derivatives [17]. Natural products remain an invaluable source of drug design because of their highly diversified and biologically relevant pharmacophore patterns that are particularly relevant in treating infectious diseases [18, 19]. Our aim in this study was to screen a repertoire of natural chemotypes to identify a novel scaffold with the potential to treat *C*. *difficile* infections. Herein, we utilized a whole cell, phenotypic, high-throughput screening (HTS) technology to screen the AnalytiCon MEGx library which consists of 1,000 natural products derived from plants and microorganisms. Our screening assay identified 9 molecules that possess potent anti *C*. *difficile* activity. *In vitro* susceptibility of the pathogen along with the killing kinetics, anti-toxin activity, and cytotoxic potential of the compounds were determined. The natural products were further evaluated in an acute-phase infection model of CDI in mice.

## 2. Materials and methods

### 2.1. Bacterial strains, reagents, and cell line

Bacterial strains were obtained from the same sources mentioned in a previous study (S2 Table) [20]. The bacterial strains were grown in supplemented brain heart infusion broth (BHIS) or agar at 37°C in an anaerobic chamber (Coy Laboratories) [20]. The Caco-2 cell line (American Type Culture Collection [ATCC], phosphate-buffered saline (PBS), Dulbecco’s modified Eagle’s medium (DMEM), fetal bovine serum (FBS), non-essential amino acids (NEAA), penicillin/ streptomycin, and MTS 3-(4,5-dimethylthiazol-2-yl)-5-(3-carboxymethoxyphenyl)-2-(4-sulfophenyl)-2*H*-tetrazolium) were all obtained from commercial vendors.

### 2.2. Whole cell phenotypic HTS assay

The MEGx library (AnalytiCon Discovery, Postdam, Germany) consisting of 1,000 natural products was screened as described before [20]. Briefly, the library was screened (screening concentration-3 *μ*M) by dispensing 180 nL of a 1 mM stock solution into 384-well plates using an Echo acoustic dispenser. Vancomycin (Gold Biotechnology, Olivette, MO), metronidazole (Alfa Aesar), and fidaxomicin (Cayman Chemicals, Ann Arbor, MI) (screening concentration- 10 *μ*M) were used as positive controls along with DMSO at the same concentration which served as the negative control. Data were recorded as mentioned previously [20]. The Z’ value for the assay was calculated as per the equation Z’ = 1-[(3*σ*_p_+ 3*σ*_n_)/(μ_p_- μ_n_)], where *σ* is the standard deviation, μ is the mean, p is the antibiotic-treated control, and n indicates the DMSO negative control. The plates with Z’<0.5 were repeated [21]. The inhibition of *C*. *difficile* ATCC BAA 1870 growth (%) was plotted using GraphPad Prism software version 8.0.

### 2.3. *In vitro* susceptibility assay

Natural products that exhibited ≥95% inhibition of *C*. *difficile* were purchased in a larger quantity from AnalytiCon Discovery. The minimum inhibitory concentration (MIC) values of natural products and antibiotics (fidaxomicin and vancomycin) were assessed as described before [22–26]. The MIC_50_ and MIC_90_ values reported are the lowest concentration of each natural product/control antibiotic that could inhibit bacterial growth by 50% and 90%, respectively.

### 2.4. Minimum bactericidal concentration (MBC) assay

The MBC values of the natural products were determined. After determining the MIC, aliquots were taken from each well in the plate that showed no visible growth and were plated on BHIS agar and incubated anaerobically to determine the MBC. The highest dilution of the compound that showed no growth was categorized as the MBC [24, 27].

### 2.5. Time-kill kinetics study

Pre-reduced BHIS medium was inoculated with *C*. *difficile* ATCC BAA 1870. Following an overnight incubation period, cultures were diluted 1:50 into fresh BHIS broth (~10^6^ CFU/ml) and added to tubes containing either DMSO, one of the natural product compounds (5 × MIC), or the control antibiotics vancomycin or fidaxomicin (5 × MIC). Viable counts of bacterial colonies were determined by taking aliquots at 0, 2-, 4-, 8-, 12-, and 24-hours post inoculation, serially diluting, and plating on BHIS agar [28, 29]. The plates were incubated overnight and CFU count for different time points were recorded the following day.

### 2.6. Toxin inhibition assay

The ability of the natural products to inhibit toxin production by the pathogen was evaluated as described before [22–24]. Briefly, (~10^6^ CFU/ml) *C*. *difficile* ATCC BAA 1870 was incubated overnight with 0.5 × MIC and 0.25 × MIC of the natural product compounds or control antibiotics. After incubation, the suspension was evaluated for the bacterial count as well as the presence of toxins (tcdA and tcdB) using an enzyme- linked immunosorbent assay (ELISA) kit (tgc BIOMICS). The OD_450_ was measured using a BioTek Gen 5 spectrophotometer.

### 2.7. Cytotoxicity assay

A cytotoxicity assay against Caco-2 cells was performed to assess the potential toxic effect of the natural products as described before [20, 30–32]. Caco-2 cells cultured in DMEM media supplemented with 10% FBS, 1% NEAA, and 1% penicillin/streptomycin were grown at 37°C in presence of 5% CO_2_. Following trypsinization, cells were seeded to 96-well plates and allowed to grow till cells in each well attained 100% confluence. Hit natural products were added to the cells at a starting concentration of 16 μg/ml whereas control wells received DMSO at the same concentration. The plates were incubated for 24 hrs. after which MTS reagent was added and absorbance was recorded at 490 nm using a SpectraMax i3Multi-Mode Microplate Reader. Caco-2 cell survival post treatment with natural products were plotted as percentage viability of natural product-treated cells in comparison to the DMSO-treated cells using GraphPad prism v. 8.0.

### 2.8. Murine model of CDI

Animal experiments were approved by the Purdue Animal Care and Use Committee (Protocol #: 1704001567) and were conducted in accordance with the National Institutes of Health Guide for the Care and Use of Laboratory Animals. The weight of each mouse and the development of CDI symptoms were monitored every 4 hours following infection and all efforts were made to minimize their suffering. Briefly, 6-week-old female C57BL/6 mice (The Jackson Laboratory) were randomly assigned to groups (n = 5). Mice were given autoclaved water and food. For the *C*. *difficile* primary infection model [29, 32–34], mice were provided with antibiotic water for 5 days. The antibiotic water consisted of colistin (850 U/ml), kanamycin (0.4 mg/ml), gentamicin (0.035 mg/ml), vancomycin (0.045 mg/ml), and metronidazole (0.215 mg/ml). All mice were subsequently provided with autoclaved water for 2 days. An intraperitoneal dose of clindamycin (10 mg/kg) was administered 1 day prior to mice being orally infected with 10^6^ CFU spores of *C*. *difficile* (ATCC 43255). Treatment with vehicle (PBS), vancomycin (10 mg/kg), NP-002327 (5 mg/kg), NP-003875 (5 mg/kg), NP-004604 (5 mg/kg), and NP-009247 (5 mg/kg) was initiated 2 hours following infection. Animals that ended up losing >20% of their body weight or became moribund were euthanized.

## 3. Results

### 3.1. High-throughput screen of AnalytiCon MEGx library and *in vitro* susceptibility of *C*. *difficile* isolates to the active natural product compounds

**Fig 1:**
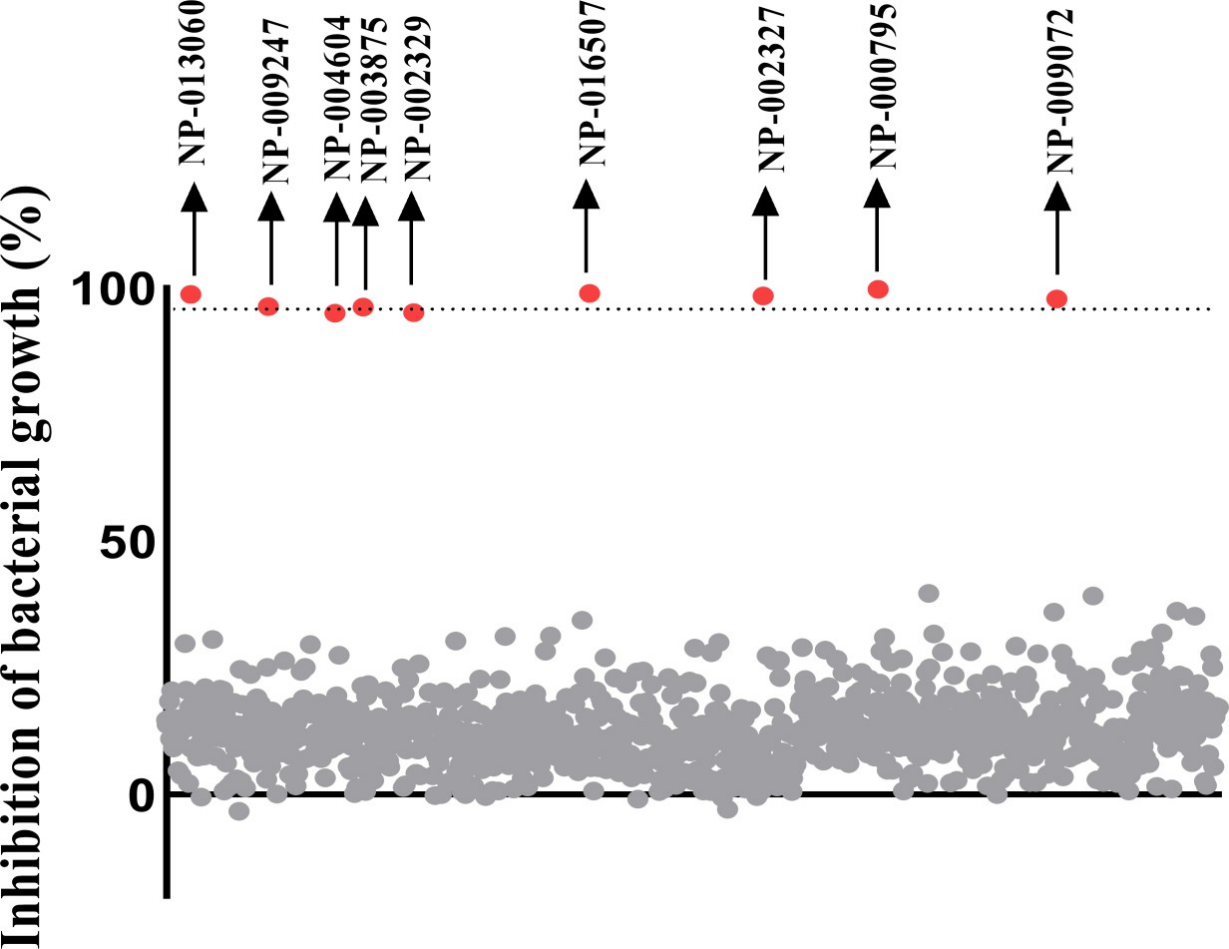
Data from the high-through screening (HTS) of the AnalytiCon MEGx library. The natural products were screened against *C*. *difficile* at 3 μM. The natural product chemotypes that exhibited greater than or equal to 95% bacterial growth inhibition were deemed as hits. The HTS assay identified 9 hits.

The AnalytiCon MegX library (1,000 compounds) was screened at 3 *μ*M to identify possible inhibitors of *C*. *difficile*. The screening assay revealed 9 compounds that inhibited the growth of the pathogen (**Fig 1 and S1 Table**).

The susceptibility of *C*. *difficile* isolates to the natural product compounds *in vitro* was determined. As depicted in **Table 1**, NP-002327, NP-002329, NP-003875, NP-004604, NP-009247, and NP-013060 displayed potent activity with MIC values that ranged between 0.03 to 2 μg/ml against all tested *C*. *difficile* strains. The MIC_50_ and MIC_90_ values for NP-003875 were found to be 0.03 μg/ml respectively. The MIC_50_ and MIC_90_ values for NP-004604 and NP-009247 were found to be 0.5 μg/ml respectively. The MIC_50_ and MIC_90_ values for NP-002327 were found to be 1 μg/ml respectively. The MIC_50_ and MIC_90_ values for NP-002329 and NP-013060 were found to be 2 μg/ml respectively. NP-000795 and NP-009072 inhibited bacterial growth at concentrations that ranged from 4 to >8 μg/ml.

**Table 1:** Minimum Inhibitory Concentration (MIC) values of natural products against a panel of clinical and hypervirulent strains of *C*. *difficile*.

*C*. *difficile* strains	NR number	MIC (*μ*g/ml)									
		NP-000795	NP-002327	NP-002329	NP-003875	NP-004604	NP-009072	NP-009247	NP-013060	Vancomycin	Fidaxomicin
I2	NR-13428	>8	1	1	0.03	0.5	4	0.25	2	1	0.03
I4	NR-13430	>8	1	2	0.03	0.5	4	0.25	1	1	0.015
I6	NR-13432	>8	2	1	0.03	0.5	>8	0.5	2	0.25	0.06
I13	NR-13553	>8	1	2	0.03	0.5	>8	0.25	2	0.25	0.03
P6	NR-32886	>8	1	1	0.03	0.5	8	0.25	1	0.125	0.03
P7	NR-32887	8	1	1	0.03	0.5	>8	0.25	2	0.5	0.03
P9	NR-32889	>8	2	2	0.015	0.5	>8	0.5	2	1	0.03
P19	NR-32895	>8	2	2	0.015	0.5	8	0.5	2	1	0.03
P30	NR-32904	>8	1	1	0.015	0.5	>8	0.25	1	0.25	0.06
Isolate 20100502	NR-49277	>8	2	2	0.03	0.5	>8	0.5	2	0.25	0.06
Isolate 20100207	NR-49278	>8	1	2	0.03	0.5	>8	0.5	2	0.25	0.125
Isolate 20110999	NR-49286	>8	1	2	0.03	0.5	>8	0.5	2	0.25	0.25
Isolate 20110870	NR-49288	>8	1	2	0.03	0.5	>8	0.5	2	1	0.125
Isolate 20120187	NR-49290	>8	1	2	0.03	0.5	>8	0.5	2	1	0.06
ATCC BAA 1870		>8	1	2	0.03	0.5	>8	0.5	2	1	0.06
ATCC 43255		>8	1	2	0.03	0.5	>8	0.5	2	1	0.06
**MIC**_**50**_		>8	1	2	0.03	0.5	>8	0.5	2	0.5	0.06
**MIC**_**90**_		>8	1	2	0.03	0.5	>8	0.5	2	1	0.125

### 3.2. MBC values and time-kill kinetics

The MBC values for the active compounds against each *C*. *difficile* strain are shown in **Table 2**. The natural product compounds demonstrated bactericidal activity against *C*. *difficile* isolates, similar to the control antibiotics fidaxomicin and vancomycin.

**Table 2:** Minimum Bactericidal Concentration (MBC) values of natural products against a panel of clinical and hypervirulent strains of *C*. *difficile*.

*C*. *difficile* strains	NR number	MBC (*μ*g/ml)							
		NP-002327	NP-002329	NP-003875	NP-004604	NP-009247	NP-013060	Vancomycin	Fidaxomicin
I2	NR-13428	1	1	0.03	0.5	0.25	2	1	0.03
I4	NR-13430	1	2	0.03	0.5	0.25	1	1	0.015
I6	NR-13432	2	1	0.03	0.5	0.5	2	0.25	0.06
I13	NR-13553	1	2	0.03	0.5	0.25	2	0.25	0.03
P6	NR-32886	1	1	0.03	0.5	0.25	1	0.125	0.03
P7	NR-32887	1	1	0.03	0.5	0.25	2	0.5	0.03
P9	NR-32889	2	2	0.015	0.5	0.5	2	1	0.03
P19	NR-32895	2	2	0.015	1	0.5	2	2	0.125
P30	NR-32904	1	1	0.015	0.5	0.25	1	0.25	0.06
Isolate 20100502	NR-49277	2	2	0.03	0.5	0.5	2	0.25	0.06
Isolate 20100207	NR-49278	1	4	0.06	0.5	1	2	0.5	0.125
Isolate 20110999	NR-49286	1	2	0.03	0.5	0.5	2	0.25	0.25
Isolate 20110870	NR-49288	1	2	0.03	1	0.5	2	1	0.125
Isolate 20120187	NR-49290	1	2	0.03	0.5	0.5	2	1	0.06
ATCC BAA 1870		2	2	0.03	0.5	0.5	2	4	0.125
ATCC 43255		1	2	0.03	0.5	0.5	2	1	0.06

**Fig 2:**
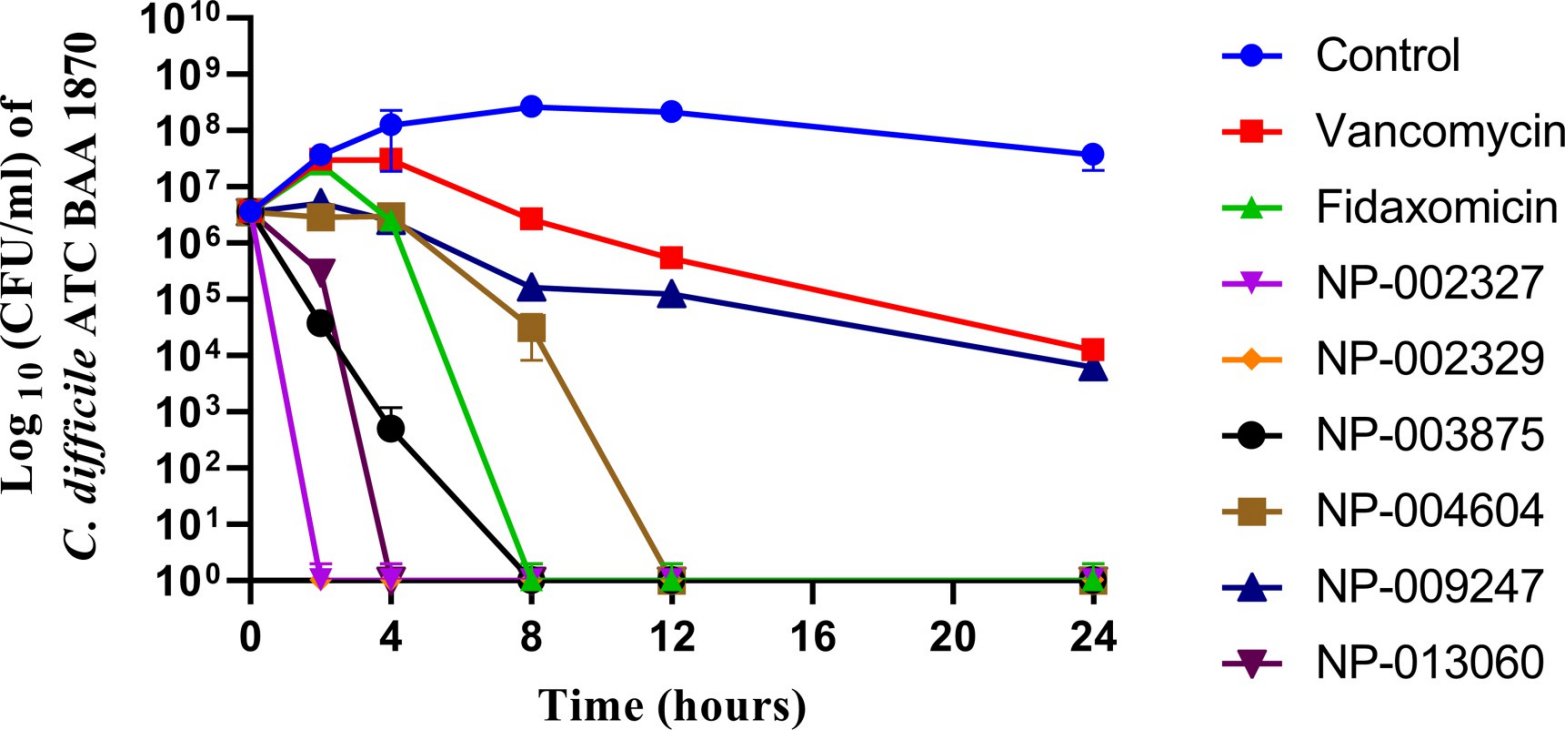
Killing kinetics of hit natural products, vancomycin, and fidaxomicin (5X MIC) against *C*. *difficile*. The standard deviation values for the triplicate samples of each natural product/control antibiotic have been represented by error bars.

The comparative killing kinetics of the natural product compounds (NP-002327, NP-002329, NP-003875, NP-004604, NP-009247, and NP-013060), vancomycin, and fidaxomicin, all tested at 5 × MIC, were assessed against *C*. *difficile* ATCC BAA 1870 as shown in **Fig 2**. The starting inoculum for the time-kill assay was 3.5 × 10^6^ cfu/ml. Fidaxomicin achieved bactericidal activity, defined as ≥ -3log_10_ CFU/ml reduction in bacterial viability, within 8 h of exposure. Vancomycin achieved a ~2.75-log_10_ CFU/ml reduction in bacterial count by 24 h. NP-002327 and NP-002329 rapidly reduced cell viability below the limit of detection (LOD) within 2 h of exposure while NP-013060 reduced cell viability below the LOD within 4 h of exposure. NP-003875 and NP-004604 reduced cell viability below the LOD within 8 and 12 h of exposure respectively. NP-009247 achieved a ~3.45-log_10_ CFU/ml reduction in bacterial count by 24 h.

### 3.3. Toxin inhibition assay

**Fig 3:**
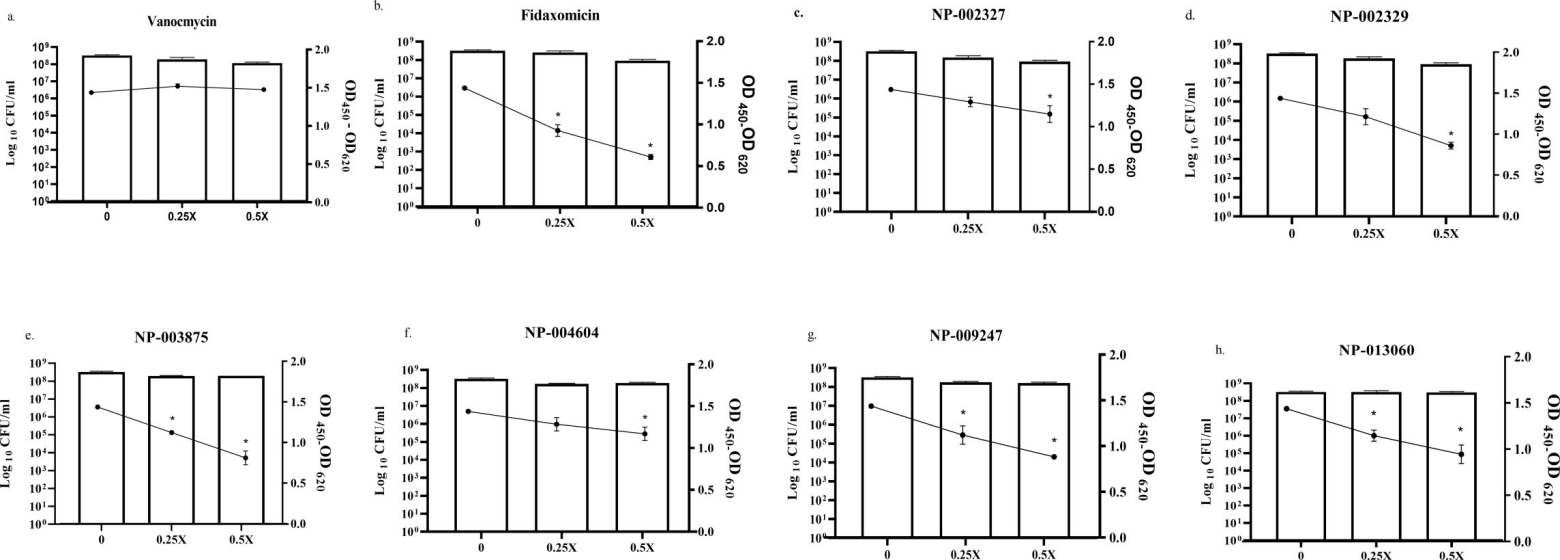
*C*. *difficile* toxin inhibition by natural products and control antibiotics (vancomycin and fidaxomicin). *C*. *difficile* was incubated with subinhibitory concentrations of drugs for 12 hours. Viable cells (log_10_ CFU/ml, bars) in each sample were determined by plating and the amount of toxin in each supernatant (OD_450_-OD_620_, lines) was assessed using ELISA. The data represents the mean and standard deviation for triplicate samples of each treatment. Asterisk (*) indicates a statically significant difference in the toxin content of the supernatant between the fidaxomicin- or natural product-treated samples and the untreated control.

As depicted in **Fig 3**, addition of subinhibitory concentrations (0.25 × MIC and 0.5 × MIC) of the natural product compounds did not affect bacterial cell viability but demonstrated an inhibitory effect on toxin production. Toxin production was reduced by approximately 10% (at 0.25 × MIC) and 20% (at 0.5 × MIC) by NP-002327, by 15% (at 0.25 × MIC) and 40% (at 0.5 × MIC) by NP-002329, by 29% (at 0.25 × MIC) and 40% (at 0.5 × MIC) by NP-003875, by 6% (at 0.25 × MIC) and 22% (at 0.5 × MIC) by NP-004604, by 22% (at 0.25 × MIC) and 38% (at 0.5 × MIC) by NP-009247, and by 20% (at 0.25 × MIC) and 34% (at 0.5 × MIC) by NP-013060. Of the control antibiotics, vancomycin did not reduce toxin production whereas fidaxomicin demonstrated an approximate 36% (at 0.25 × MIC) and 57% (at 0.5 × MIC) reduction in toxin production.

### 3.4. Cytotoxicity assay

**Fig 4:**
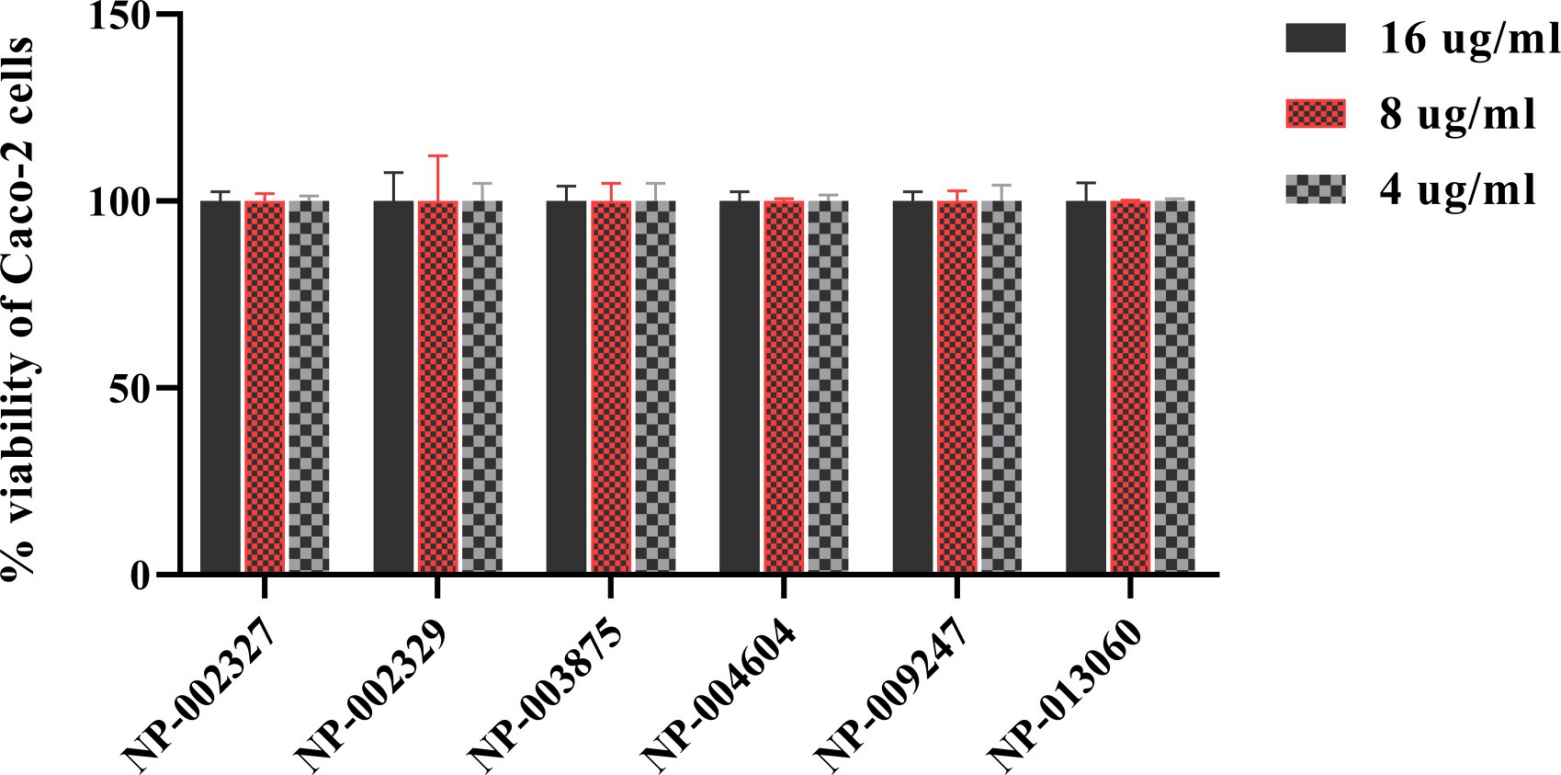
Cytotoxicity assay of natural products against Caco-2 cell line. The percentage of viable Caco-2 cells was measured as the ratio of average absorbance of each sample relative to the negative control (DMSO). The absorbance values represent the mean of three samples of each natural product compound with error bars representing standard deviation values for the absorbance.

The cytotoxic effect of the compounds was evaluated against Caco-2 cells using an MTS assay. **Fig 4** presents the results of the cytotoxicity assay. All 6 natural product compounds evaluated (NP-002327, NP-002329, NP-003875, NP-004604, NP-009247, and NP-013060) did not exhibit toxicity to Caco-2 cells at a concentration up to 16 μg/ml.

### 3.5. Murine model of CDI

**Fig 5:**
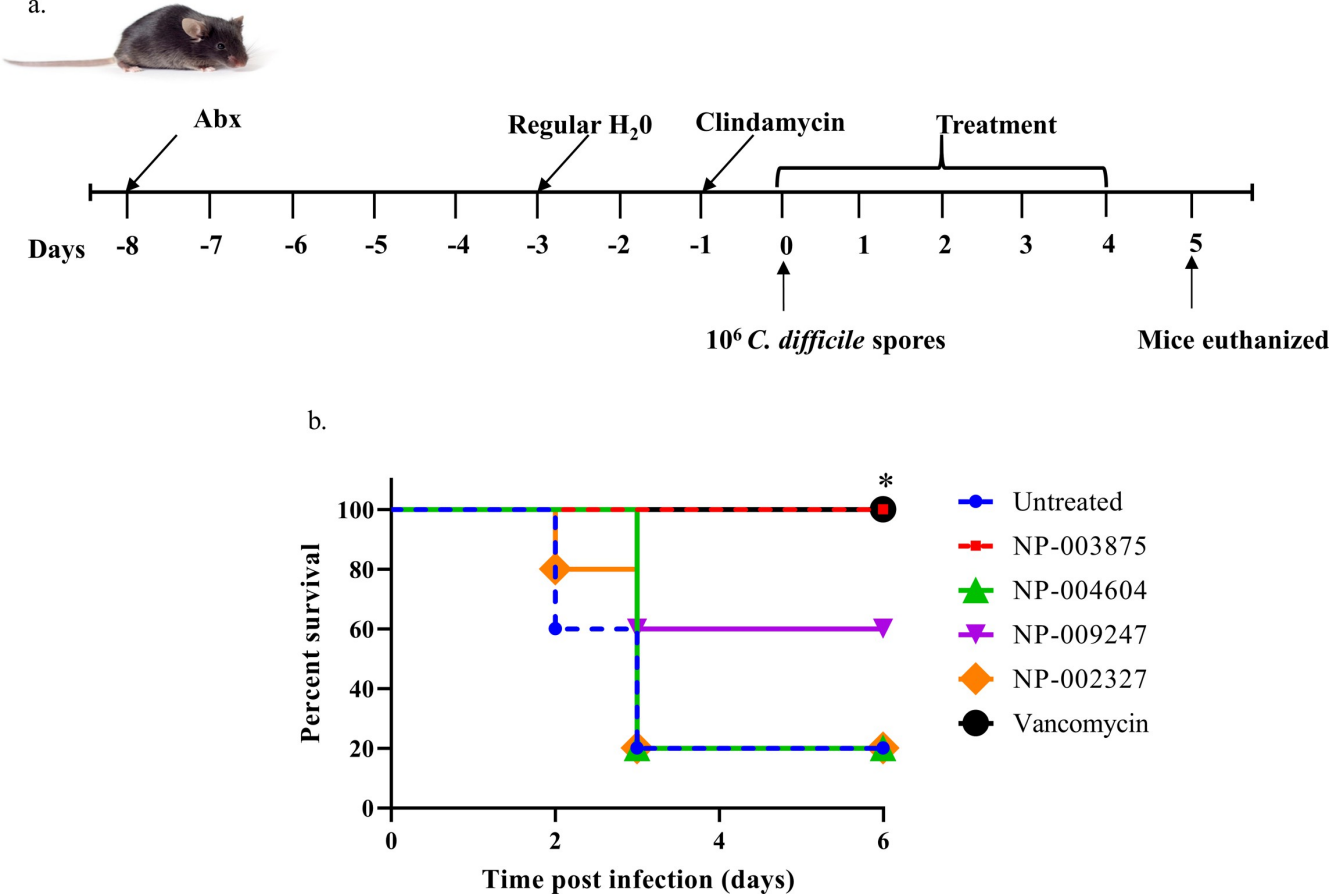
Murine model of CDI. **(A)** Schematic of mice model. 6-week-old female mice were randomly divided into groups (n = 5 per group). Mice were given antibiotic water consisting of kanamycin (0.4 mg/ml), colistin (850 U/ml), gentamicin (0.035 mg/ml), vancomycin (0.045 mg/ml), and metronidazole (0.215 mg/ml) for 5 days. Clindamycin (10 mg/kg) was injected intraperitoneally a day before infection. *C*. *difficile* spores were administered via oral gavage on Day 0. Treatment was initiated on Day 0 and continued for 5 days. A single dose of each drug was administered via oral gavage daily. **(B)** The Kaplan-Meier survival curve was analyzed using a log-rank (Mantel-Cox) test. Significant difference was noted between the survival curves of the mice treated with NP-003875 vs. untreated control (100% vs. 20%; p = 0.0144).

For a more clinically relevant test of the potency of the compounds, the *in vivo* effect of 4 natural product compounds with the lowest MIC values *in vitro* (NP-002327, NP-003875, NP-004604, and NP-009247) were investigated in a murine model of an acute-phase model of CDI (**Fig 5**). Mice infected with *C*. *difficile* spores were treated with vehicle, one of the natural product compounds, or vancomycin (10 mg/kg). Of the 4 natural products, NP-003875 was found to confer protection to 100% of the mice at 5 mg/kg dose, similar to that of the control antibiotic vancomycin which was administered at a 10 mg/kg dose. NP-009247 protected 60% of the mice whereas NP-002327 and NP-004604 failed to confer any protection at all.

## 4. Discussion

*C*. *difficile* is an enteric pathogen that continues to affect patients in hospitals and communities globally. The opportunistic pathogen exploits a reduction in gut microflora following the use of broad-spectrum antibiotics and causes life-threatening colitis [35]. The current treatment regimen for CDI consists of two antibiotics, vancomycin and fidaxomicin. These standard-of-care antibiotics fail to achieve a complete clinical cure and recurrence of infection is of common occurrence in CDI patients [36]. In the face of the significant drawbacks associated with the current pharmaceutical armamentarium, alternative options for treating CDI are urgently needed.

Natural products have been played a pivotal role in drug discovery. The natural product pool is rich in bioactive compounds characterized by diversified scaffolds unlike libraries consisting of conventional synthetic molecules [37]. Here, our aim was to identify anticlostridial leads from a library of extracts from natural sources (plants and microorganisms). Consequently, we conducted a whole cell phenotypic HTS of the AnalytiCon MEGx library (1,000 natural products) and initially identified 9 natural compounds that could inhibit the growth of *C*. *difficile* at a concentration of 3 μM. Our next step was to determine the lowest concentration that the natural products could inhibit growth of this pathogen. We conducted an assay that found 6 natural products possessed MIC values that ranged from 0.03 to 2 μg/ml. Of these 6 hits, one compound (NP-003875) exhibited a 3-fold lower MIC_90_ value compared to that of fidaxomicin and 3 other hits (NP-002327, NP-004604, and NP-009247) were found to be as potent as that of vancomycin in inhibiting the growth of the pathogen. NP-002329 and NP-013060 possessed MIC values that were only one-fold higher than vancomycin.

In addition to potent growth inhibition, all 6 natural products (NP-002327, NP-002329, NP-003875, NP-004604, NP-009247, and NP-013060) exhibited bactericidal activity against *C*. *difficile*. Based on the data from the time-kill kinetic studies against *C*. *difficile* ATCC BAA 1870, all 6 hit natural product compounds exhibited more rapid bactericidal activity compared to vancomycin. Furthermore, one natural product (NP-003875) exhibited killing kinetics comparable to that of fidaxomicin. Three compounds (NP-002327, NP-002329, and NP-013060) exhibited more rapid bactericidal activity compared to fidaxomicin. This rapid bactericidal activity can be of potential importance clinically as it can reduce the emergence of resistant bacterial strains and can rapidly resolve infection and associated clinical signs [38].

Extensive epidemiological and experimental evidence support the role of *C*. *difficile* toxins in driving disease pathogenesis. Isogeneic knockout studies have shown that either of TcdA or TcdB alone can cause fulminant colitis in hamsters [39, 40]. Of the clinically used antibiotics, fidaxomicin is the only drug that can inhibit toxin production by the pathogen [41]. This paucity of drugs with antitoxin activity has fueled efforts to identify molecules that can inhibit *C*. *difficile* toxins. Therefore, we investigated the ability of our natural product compounds as a potential antivirulence therapeutic agent. The 6 natural products, at 0.5 × MIC, reduced toxin production by *C*. *difficile* ATCC BAA 1870 unlike vancomycin which had no inhibitory effect on toxin production. Fidaxomicin, on the other hand, demonstrated a 57% reduction in *C*. *difficile* toxin production at 0.5 × MIC.

*In vitro* cytotoxicity testing is a pivotal step for consideration of compounds in drug discovery [42]. The cytotoxicity assay of the 6 active natural products against Caco-2 cells revealed no detrimental effects on the human colorectal cells when treated at a concentration of 16 *μ*g/ml for 24 hours.

To further elucidate the potency of the compounds and determine their clinical relevance in the treatment of CDI, we sought to test the efficacy of the natural products in an acute-phase model of CDI infection in mice. Interestingly, 1 natural product (NP-003875) protected 100% of mice from the clinical manifestations of CDI similar to the standard-of-care antibiotic vancomycin.

In conclusion, we identified a novel lead compound, NP-003875, with potent *in vitro* and *in vivo* anticlostridial activity. Structural interpretation of NP-003875 led to its identification as chromomycin A2, a member of the aureolic acid family of bacterial natural products isolated from *Streptomyces* sp. Like its previously studied structural analogue mithramycin [22], chromomycin A2 harbors potent anticlostridial activity inhibiting *C*. *difficile in vitro* at a concentration of 0.03 μg/ml, is bactericidal, and exhibits time-kill kinetics similar to that of fidaxomicin. The mode of action of the members of the aureolic acid family involves interaction with the DNA helix minor groove causing a DNA-dependent inhibition of RNA synthesis [43]. As observed with mithramycin, chromomycin A2 can also inhibit *C*. *difficile* toxins, hinting towards a potential similar mechanism of action based on their structural similarities. In addition, chromomycin A2/NP-003875 was found to be non-toxic to Caco-2 cells and was determined to be as efficacious as vancomycin in protecting mice infected with *C*. *difficile*. Further studies, including validating its mechanism of action, investigating NP-003875’s ability to inhibit spore formation by *C*. *difficile* and determining its efficacy in a recurrent infection model of the pathogen will be needed to evaluate the potency of this compound in ameliorating the symptoms of CDI.

## Supporting information

S1 Table Structural and IUPAC names of hit natural products. (DOCX)

S2 Table List of *C*. *difficile* strains used in the study. (DOCX)

S1 Data (PZFX)

S2 Data (PZFX)

S3 Data (PZFX)

S1 File (DOCX)

## References

[1] Redelings MD, Sorvillo F, Mascola L. Increase in Clostridium difficile-related mortality rates, United States, 1999–2004. Emerg Infect Dis. 2007;13(9):1417–9. Epub 2008/02/07. doi: 10.3201/eid1309.061116 ; PubMed Central PMCID: PMC2857309. 18252127 PMC2857309

[2] Leffler DA, Lamont JT. Clostridium difficile infection. N Engl J Med. 2015;372(16):1539–48. doi: 10.1056/NEJMra1403772 . 25875259

[3] CDC. Antibiotic Resistance Threats in the United States, 2019. Atlanta, GA: U.S. Department of Health and Human Services, CDC. 2019.

[4] Theriot CM, Bowman AA, Young VB. Antibiotic-Induced Alterations of the Gut Microbiota Alter Secondary Bile Acid Production and Allow for Clostridium difficile Spore Germination and Outgrowth in the Large Intestine. mSphere. 2016;1(1). Epub 2016/05/31. doi: 10.1128/mSphere.00045-15 ; PubMed Central PMCID: PMC4863611. 27239562 PMC4863611

[5] Paparella AS, Aboulache BL, Harijan RK, Potts KS, Tyler PC, Schramm VL. Inhibition of Clostridium difficile TcdA and TcdB toxins with transition state analogues. Nat Commun. 2021;12(1):6285. Epub 20211101. doi: 10.1038/s41467-021-26580-6 ; PubMed Central PMCID: PMC8560925. 34725358 PMC8560925

[6] Rupnik M, Wilcox MH, Gerding DN. Clostridium difficile infection: new developments in epidemiology and pathogenesis. Nat Rev Microbiol. 2009;7(7):526–36. Epub 2009/06/17. doi: 10.1038/nrmicro2164 . 19528959

[7] Kordus SL, Thomas AK, Lacy DB. Clostridioides difficile toxins: mechanisms of action and antitoxin therapeutics. Nat Rev Microbiol. 2021. Epub 20211126. doi: 10.1038/s41579-021-00660-2 . 34837014 PMC9018519

[8] Hryckowian AJ, Pruss KM, Sonnenburg JL. The emerging metabolic view of Clostridium difficile pathogenesis. Curr Opin Microbiol. 2017;35:42–7. Epub 2016/12/21. doi: 10.1016/j.mib.2016.11.006 ; PubMed Central PMCID: PMC5474191. 27997854 PMC5474191

[9] El Feghaly RE, Stauber JL, Deych E, Gonzalez C, Tarr PI, Haslam DB. Markers of intestinal inflammation, not bacterial burden, correlate with clinical outcomes in Clostridium difficile infection. Clin Infect Dis. 2013;56(12):1713–21. Epub 20130313. doi: 10.1093/cid/cit147 ; PubMed Central PMCID: PMC3707425. 23487367 PMC3707425

[10] Shim JK, Johnson S, Samore MH, Bliss DZ, Gerding DN. Primary symptomless colonisation by Clostridium difficile and decreased risk of subsequent diarrhoea. Lancet. 1998;351(9103):633–6. Epub 1998/03/21. doi: 10.1016/S0140-6736(97)08062-8 . 9500319

[11] Kelly CP. Can we identify patients at high risk of recurrent Clostridium difficile infection? Clin Microbiol Infect. 2012;18 Suppl 6:21–7. Epub 2012/11/21. doi: 10.1111/1469-0691.12046 . 23121551

[12] Kuehne SA, Cartman ST, Heap JT, Kelly ML, Cockayne A, Minton NP. The role of toxin A and toxin B in Clostridium difficile infection. Nature. 2010;467(7316):711–3. Epub 20100915. doi: 10.1038/nature09397 . 20844489

[13] McDonald LC, Gerding DN, Johnson S, Bakken JS, Carroll KC, Coffin SE, et al. Clinical Practice Guidelines for Clostridium difficile Infection in Adults and Children: 2017 Update by the Infectious Diseases Society of America (IDSA) and Society for Healthcare Epidemiology of America (SHEA). Clin Infect Dis. 2018;66(7):e1–e48. Epub 2018/02/21. doi: 10.1093/cid/cix1085 ; PubMed Central PMCID: PMC6018983. 29462280 PMC6018983

[14] McGovern BH, Ford CB, Henn MR, Pardi DS, Khanna S, Hohmann EL, et al. SER-109, an Investigational Microbiome Drug to Reduce Recurrence After Clostridioides difficile Infection: Lessons Learned From a Phase 2 Trial. Clin Infect Dis. 2021;72(12):2132–40. doi: 10.1093/cid/ciaa387 ; PubMed Central PMCID: PMC8204772. 32255488 PMC8204772

[15] Gerber M, Walch C, Loffler B, Tischendorf K, Reischl U, Ackermann G. Effect of sub-MIC concentrations of metronidazole, vancomycin, clindamycin and linezolid on toxin gene transcription and production in Clostridium difficile. J Med Microbiol. 2008;57(Pt 6):776–83. Epub 2008/05/16. doi: 10.1099/jmm.0.47739-0 . 18480337

[16] Harvey AL, Edrada-Ebel R, Quinn RJ. The re-emergence of natural products for drug discovery in the genomics era. Nat Rev Drug Discov. 2015;14(2):111–29. Epub 2015/01/24. doi: 10.1038/nrd4510 . 25614221

[17] Newman DJ, Cragg GM. Natural Products as Sources of New Drugs over the Nearly Four Decades from 01/1981 to 09/2019. J Nat Prod. 2020;83(3):770–803. Epub 2020/03/13. doi: 10.1021/acs.jnatprod.9b01285 . 32162523

[18] Rodrigues T, Reker D, Schneider P, Schneider G. Counting on natural products for drug design. Nat Chem. 2016;8(6):531–41. Epub 2016/05/25. doi: 10.1038/nchem.2479 . 27219696

[19] Patridge E, Gareiss P, Kinch MS, Hoyer D. An analysis of FDA-approved drugs: natural products and their derivatives. Drug Discov Today. 2016;21(2):204–7. Epub 2015/01/27. doi: 10.1016/j.drudis.2015.01.009 . 25617672

[20] Pal R, Dai M, Seleem MN. High-throughput screening identifies a novel natural product-inspired scaffold capable of inhibiting Clostridioides difficile in vitro. Sci Rep. 2021;11(1):10913. Epub 2021/05/27. doi: 10.1038/s41598-021-90314-3 ; PubMed Central PMCID: PMC8149678. 34035338 PMC8149678

[21] Zhang J-H, Chung TD, Oldenburg KR. A simple statistical parameter for use in evaluation and validation of high throughput screening assays. Journal of biomolecular screening. 1999;4(2):67–73. doi: 10.1177/108705719900400206 10838414

[22] Pal R, Seleem MN. Screening of Natural Products and Approved Oncology Drug Libraries for Activity against Clostridioides difficile. Sci Rep. 2020;10(1):5966. Epub 2020/04/07. doi: 10.1038/s41598-020-63029-0 ; PubMed Central PMCID: PMC7136261. 32249833 PMC7136261

[23] AbdelKhalek A, Abutaleb NS, Mohammad H, Seleem MN. Antibacterial and antivirulence activities of auranofin against Clostridium difficile. Int J Antimicrob Agents. 2019;53(1):54–62. Epub 2018/10/03. doi: 10.1016/j.ijantimicag.2018.09.018 ; PubMed Central PMCID: PMC6475173. 30273668 PMC6475173

[24] Abutaleb NS, Seleem MN. Repurposing the Antiamoebic Drug Diiodohydroxyquinoline for Treatment of Clostridioides difficile Infections. Antimicrob Agents Chemother. 2020;64(6). Epub 2020/04/08. doi: 10.1128/AAC.02115-19 ; PubMed Central PMCID: PMC7269495. 32253206 PMC7269495

[25] Shao X, AbdelKhalek A, Abutaleb NS, Velagapudi UK, Yoganathan S, Seleem MN, et al. Chemical Space Exploration around Thieno[3,2-d]pyrimidin-4(3H)-one Scaffold Led to a Novel Class of Highly Active Clostridium difficile Inhibitors. J Med Chem. 2019;62(21):9772–91. Epub 2019/10/05. doi: 10.1021/acs.jmedchem.9b01198 . 31584822

[26] Mody D, Athamneh AIM, Seleem MN. Curcumin: A natural derivative with antibacterial activity against Clostridium difficile. J Glob Antimicrob Resist. 2020;21:154–61. Epub 2019/10/18. doi: 10.1016/j.jgar.2019.10.005 ; PubMed Central PMCID: PMC7153983. 31622683 PMC7153983

[27] Basseres E, Begum K, Lancaster C, Gonzales-Luna AJ, Carlson TJ, Miranda J, et al. In vitro activity of eravacycline against common ribotypes of Clostridioides difficile. J Antimicrob Chemother. 2020;75(10):2879–84. Epub 2020/07/29. doi: 10.1093/jac/dkaa289 ; PubMed Central PMCID: PMC7678891. 32719870 PMC7678891

[28] Corbett D, Wise A, Birchall S, Warn P, Baines SD, Crowther G, et al. In vitro susceptibility of Clostridium difficile to SMT19969 and comparators, as well as the killing kinetics and post-antibiotic effects of SMT19969 and comparators against C. difficile. J Antimicrob Chemother. 2015;70(6):1751–6. Epub 2015/02/06. doi: 10.1093/jac/dkv006 ; PubMed Central PMCID: PMC4498293. 25652750 PMC4498293

[29] AbdelKhalek A, Seleem MN. Repurposing the Veterinary Antiprotozoal Drug Ronidazole for the Treatment of Clostridioides difficile Infection. Int J Antimicrob Agents. 2020;56(6):106188. Epub 2020/10/13. doi: 10.1016/j.ijantimicag.2020.106188 ; PubMed Central PMCID: PMC7704610. 33045352 PMC7704610

[30] Hamann HJ, Abutaleb NS, Pal R, Seleem MN, Ramachandran PV. beta,gamma-Diaryl alpha-methylene-gamma-butyrolactones as potent antibacterials against methicillin-resistant Staphylococcus aureus. Bioorg Chem. 2020;104:104183. Epub 2020/09/25. doi: 10.1016/j.bioorg.2020.104183 . 32971415

[31] Mohammad H, Reddy PV, Monteleone D, Mayhoub AS, Cushman M, Seleem MN. Synthesis and antibacterial evaluation of a novel series of synthetic phenylthiazole compounds against methicillin-resistant Staphylococcus aureus (MRSA). Eur J Med Chem. 2015;94:306–16. Epub 2015/03/17. doi: 10.1016/j.ejmech.2015.03.015 ; PubMed Central PMCID: PMC4716659. 25771109 PMC4716659

[32] Naclerio GA, Abutaleb NS, Li D, Seleem MN, Sintim HO. Ultrapotent Inhibitor of Clostridioides difficile Growth, Which Suppresses Recurrence In Vivo. J Med Chem. 2020;63(20):11934–44. Epub 2020/09/23. doi: 10.1021/acs.jmedchem.0c01198 . 32960605 PMC9064041

[33] Chen X, Katchar K, Goldsmith JD, Nanthakumar N, Cheknis A, Gerding DN, et al. A mouse model of Clostridium difficile-associated disease. Gastroenterology. 2008;135(6):1984–92. Epub 2008/10/14. doi: 10.1053/j.gastro.2008.09.002 . 18848941

[34] Abutaleb NS, Seleem MN. Auranofin, at clinically achievable dose, protects mice and prevents recurrence from Clostridioides difficile infection. Sci Rep. 2020;10(1):7701. Epub 2020/05/10. doi: 10.1038/s41598-020-64882-9 ; PubMed Central PMCID: PMC7206065. 32382070 PMC7206065

[35] Kirk JA, Gebhart D, Buckley AM, Lok S, Scholl D, Douce GR, et al. New class of precision antimicrobials redefines role of Clostridium difficile S-layer in virulence and viability. Sci Transl Med. 2017;9(406). Epub 2017/09/08. doi: 10.1126/scitranslmed.aah6813 ; PubMed Central PMCID: PMC5603275. 28878013 PMC5603275

[36] Lessa FC, Mu Y, Bamberg WM, Beldavs ZG, Dumyati GK, Dunn JR, et al. Burden of Clostridium difficile infection in the United States. N Engl J Med. 2015;372(9):825–34. Epub 2015/02/26. doi: 10.1056/NEJMoa1408913 . 25714160 PMC10966662

[37] Atanasov AG, Zotchev SB, Dirsch VM, International Natural Product Sciences T, Supuran CT. Natural products in drug discovery: advances and opportunities. Nat Rev Drug Discov. 2021;20(3):200–16. Epub 2021/01/30. doi: 10.1038/s41573-020-00114-z ; PubMed Central PMCID: PMC7841765. 33510482 PMC7841765

[38] Mohammad H, Mayhoub AS, Cushman M, Seleem MN. Anti-biofilm activity and synergism of novel thiazole compounds with glycopeptide antibiotics against multidrug-resistant staphylococci. J Antibiot (Tokyo). 2015;68(4):259–66. Epub 2014/10/16. doi: 10.1038/ja.2014.142 ; PubMed Central PMCID: PMC4429288. 25315757 PMC4429288

[39] Lyras D, O’Connor JR, Howarth PM, Sambol SP, Carter GP, Phumoonna T, et al. Toxin B is essential for virulence of Clostridium difficile. Nature. 2009;458(7242):1176–9. Epub 2009/03/03. doi: 10.1038/nature07822 ; PubMed Central PMCID: PMC2679968. 19252482 PMC2679968

[40] Carter GP, Rood JI, Lyras D. The role of toxin A and toxin B in Clostridium difficile-associated disease: Past and present perspectives. Gut Microbes. 2010;1(1):58–64. Epub 2010/07/29. doi: 10.4161/gmic.1.1.10768 ; PubMed Central PMCID: PMC2906822. 20664812 PMC2906822

[41] Babakhani F, Bouillaut L, Sears P, Sims C, Gomez A, Sonenshein AL. Fidaxomicin inhibits toxin production in Clostridium difficile. J Antimicrob Chemother. 2013;68(3):515–22. Epub 2012/12/05. doi: 10.1093/jac/dks450 . 23208832

[42] Niles AL, Moravec RA, Riss TL. Update on in vitro cytotoxicity assays for drug development. Expert Opin Drug Discov. 2008;3(6):655–69. Epub 2008/06/01. doi: 10.1517/17460441.3.6.655 . 23506147

[43] Lombo F, Menendez N, Salas JA, Mendez C. The aureolic acid family of antitumor compounds: structure, mode of action, biosynthesis, and novel derivatives. Appl Microbiol Biotechnol. 2006;73(1):1–14. Epub 20060930. doi: 10.1007/s00253-006-0511-6 . 17013601

