# Supplementary material for: Discovery of a novel natural product inhibitor of Clostridioides difficile with potent activity in vitro and in vivo
Source: PLoS One. 2022 Aug 8;17(8):e0267859. doi: 10.1371/journal.pone.0267859 (PMC9359557; doi:10.1371/journal.pone.0267859)
Supplement: S1 Table — (DOCX) [file pone.0267859.s001.docx]

**Table S1: Structure and source of hit natural products:**

| **ID number** | **Structure** | **Formulae** | **Mol. wt** | **Source** |
| --- | --- | --- | --- | --- |
| **NP-000795** |  | C_23_H_32_O_5_ | 388.497 | *Aspergillus insuetus* |
| **NP-002327** |  | C_21_H_30_O_2_ | 314.462 | Seed of *Anacardium occidentale* |
| **NP-002329** |  | C_22_H_30_O_3_ | 342.472 | Seed of *Anacardium occidentale* |
| **NP-003875** |  | C_59_H_86_O_26_ | 1211.3 | *Streptomyces* sp. |
| **NP-004604** |  | C_28_H_22_O_10_ | 518.468 | Unknown |
| **NP-009072** |  | C_28_H_36_O_5_ | 452.582 | *Kokoona zeylanica* |
| **NP-009247** |  | C_53_H_78_O_24_ | 1099.17 | *Streptomyces* sp. |
| **NP-013060** |  | C_24_H_26_O_6_ | 410.46 | Flower of *Cratoxylum prunifolium* |
