# Supplementary material for: Discovery of a novel natural product inhibitor of Clostridioides difficile with potent activity in vitro and in vivo
Source: PLoS One. 2022 Aug 8;17(8):e0267859. doi: 10.1371/journal.pone.0267859 (PMC9359557; doi:10.1371/journal.pone.0267859)
Supplement: S2 Table — (DOCX) [file pone.0267859.s002.docx]

**Table S2: List of *C. difficile* strains used in the study.**

| **Bacterial strains / ID number** | **Source and comments** | **Ribotype** | **Characteristics** |
| --- | --- | --- | --- |
| Isolate 2/ NR-13428 | Isolated in 2008/2009 from a patient diagnosed with CDI in the Mid-Atlantic region of the USA | N/A | N/A |
| Isolate 4/ NR-13430 | Isolated in 2008/2009 from a patient diagnosed with CDI in the Mid-Atlantic region of the USA | N/A | N/A |
| Isolate 6/ NR-13432 | Isolated 2008/2009 from a patient diagnosed with CDI in the Mid-Atlantic region of the USA | N/A | N/A |
| Isolate 13/ NR-13553 | Isolated 2008/2009 from a patient diagnosed with CDI in the Mid-Atlantic region of the USA | N/A | N/A |
| P6/ NR-32886 | Procured in 2001from fecal matter of a patient suffering from recurrent *C. difficile* infection in western Pennsylvania, USA | N/A | Toxigenic strain |
| P7/ NR-32887 | Procured in 2001from fecal matter of a patient with *C. difficile* infection in western Pennsylvania, USA | N/A | Toxigenic strain |
| P9/ NR-32889 | Procured in 2001from fecal matter of a patient suffering from recurrent *C. difficile* infection | N/A | Toxigenic strain |
| P19/ NR-32895 | Procured from fecal matter of a patient suffering from recurrent *C. difficile* infection in western Pennsylvania, USA in 2005 | N/A | Toxigenic strain |
| P30/ NR-32904 | Isolated in 2009 from fecal matter of an asymptomatic human patient in western Pennsylvania, USA | N/A | Non-toxigenic strain |
| Isolate 20100502/ NR-49277 | Isolated from the fecal matter of an elderly male patient diagnosed with community-associated (CA) *C. difficile* infection in Colorado, USA in the year of 2010 | Ribotype 19 | *tcdA*^b^*, tcdB*^c^, and cdtB^a^ |
| Isolate 20100207/ NR-49278 | Isolated from the stool of an elderly adult male patient diagnosed with healthcare-associated (HA) *C. difficile* infection in New York, USA in the year of 2010 | Ribotype 027 | *tcdA*^b^*, tcdB*^c^, and cdtB^a^ |
| Isolate 20110999/NR-49286 | Isolated from the stool sample of an elderly female patient diagnosed with healthcare-associated (HA) *C. difficile* infection in western/midwestern, USA in the year of 2011 | Ribotype 027 | *tcdA*^b^*, tcdB*^c^, and cdtB^a^ |
| Isolate 20110870/ NR-49288 | Isolated from the stool sample of a young adult female patient diagnosed with healthcare associated (HA) *C. difficile* infection in Tennessee, USA in the year of 2011 | Ribotype 027 | *tcdA*^b^*, tcdB*^c^, and cdtB^a^ |
| Isolate 20120187/ NR-49290 | Isolated from the stool sample of an elderly adult male patient with healthcare-associated (HA) *C. difficile* infection in Tennessee, USA in the year of 2011 | Ribotype 19 | *tcdA*^b^*, tcdB*^c^, and cdtB^a^ |
| ATCC BAA 1870 | N/A | Ribotype 027 | *tcdA*^b^*, tcdB*^c^, and cdtB^a^ |
| ATCC 43255/ VPI 10463 | N/A | Ribotype 087 | *tcdA*^b^ and *tcdB*^c^ |

^a^ cdtB= *C. difficile* binary toxin gene

^b^ tcdA= *C. difficile* toxin A gene

^c^ tcdB= *C. difficile* toxin B gene
